# Enhancing Surgical Wound Monitoring: A Paired Cohort Study Evaluating a New AI-Based Application for Automatic Detection of Potential Infections

**DOI:** 10.3390/jcm13247863

**Published:** 2024-12-23

**Authors:** Andrea Craus-Miguel, Marc Munar, Gabriel Moyà-Alcover, Ana María Contreras-Nogales, Manuel González-Hidalgo, Juan José Segura-Sampedro

**Affiliations:** 1 General and Digestive Surgery Department, Clínica Universidad de Navarra, 31008 Madrid, Spain; 2 PhD Program in Translational Research in Public Health and High Prevalence Diseases, Health Sciences, University of the Balearic Islands (UIB), 07122 Palma, Spain; 3 Soft Computing, Image Processing and Aggregation (SCOPIA) Research Group, University of the Balearic Islands (UIB), 07122 Palma, Spain; 4 Artificial Intelligence Research Institute of the UIB (IAIB), University of the Balearic Islands (UIB), 07122 Palma, Spain; 5 Health Research Institute of the Balearic Islands (IdISBa), 07120 Palma, Spain; 6 Computer Graphics and Vision and AI (UGiVIA) Research Group, University of the Balearic Islands (UIB), 07122 Palma, Spain; 7 Laboratory of Artificial Intelligence Applications (LAIA@UIB), University of the Balearic Islands (UIB), 07122 Palma, Spain; 8 Nursing Team, Wound Care Unit, Outpatient Department, Son Espases University Hospital, 07120 Palma, Spain; 9 General & Digestive Surgery Service, Hospital Universitario La Paz, 28046 Madrid, Spain; segusamjj@hotmail.com; 10 Hospital La Paz Institute for Health Research (IdiPAZ), 28046 Madrid, Spain; 11 School of Medicine, Universidad CEU San Pablo, 28003 Madrid, Spain

**Keywords:** surgical wound monitoring, smartphone application, artificial intelligence, telemedicine, surgical site infection (SSI), remote monitoring, digital health, postoperative care, telematic follow-up, patient satisfaction, active patient

## Abstract

**Background/Objectives:** This study assessed the feasibility and security of remote surgical wound monitoring using the RedScar© smartphone app, which employs automated diagnosis for early visual detection of infections without direct healthcare personnel involvement. Additionally, patient satisfaction with telematic care was evaluated as a secondary aim. Surgical site infection (SSI) is the second leading cause of healthcare-associated infections (HAIs), leading to prolonged hospital stays, heightened patient distress, and increased healthcare costs. **Methods:** The study employed a prospective paired-cohort and single-blinded design, with a sample size of 47 adult patients undergoing abdominal surgery. RedScar© was used for remote telematic monitoring, evaluating the feasibility and security of this approach. A satisfaction questionnaire assessed patient experience. The study protocol was registered at ClinicalTrials.gov under the identifier NCT05485233. **Results:** Out of 47 patients, 41 successfully completed both remote and in-person follow-ups. RedScar© demonstrated a sensitivity of 100% in detecting SSIs, with a specificity of 83.13%. The kappa coefficient of 0.8171 indicated substantial agreement between the application’s results and human observers. Patient satisfaction with telemonitoring was high: 97.6% believed telemonitoring reduces costs, 90.47% perceived it prevents work/school absenteeism, and 80.9% found telemonitoring comfortable. **Conclusions:** This is the first study to evaluate an automatic smartphone application on real patients for diagnosing postoperative wound infections. It establishes the safety and feasibility of telematic follow-up using the RedScar© application for surgical wound assessment. The high sensitivity suggests its utility in identifying true cases of infection, highlighting its potential role in clinical practice. Future studies are needed to address limitations and validate the efficacy of RedScar© in diverse patient populations.

## 1. Introduction

Surgical site infection (SSI) is the second cause of healthcare-associated infections (HAIs), with a prevalence of 19.6% of all HAIs in Europe [1]. SSIs are associated with significant morbidity, including prolonged postoperative hospital stays, additional surgical procedures, increased mortality rates, and an impact on patient health-related quality of life (HRQoL) [2,3]. The incidence of SSIs varies from 0.5% to 10.1%, depending on the type of surgical procedure. Delayed diagnosis of SSIs, which often occurs after patient discharge [4], exacerbates patient distress and increases healthcare costs. In Europe, the economic burden of SSIs is substantial, with direct costs estimated to range from EUR 10,443 to EUR 19,638 per infection [5]. These costs include extended hospital stays, additional treatments, and follow-up visits. Indirect costs, such as lost productivity and long-term disability, further amplify the financial impact on healthcare systems and society [6].

In recent years, the rise of new devices and smartphone apps has advanced e-health, aligning it with patients’ real-life routines for early disease detection and proactive healthcare [7,8,9,10,11]. This deepens our understanding of disease progression, resulting in reduced mortality and improved well-being. With mobile technology accessible to around 85% of the population [12], telemedicine emerges as a game-changer, enhancing routine care while concurrently diminishing wound infection occurrences and medical center visits without compromising patient satisfaction [13,14,15]. It is important to use user-friendly smartphone apps with navigation, instant assessments, and standardization, alongside predictive models powered by machine learning and artificial intelligence for better infection detection [16,17,18,19,20]

We created RedScar©, a smartphone app for remote post-discharge assessment of surgical wounds. It uses automated diagnosis to detect and evaluate surgical site infections without human involvement, enabling early identification and intervention.

Innovative Approach: This study represents a pioneering effort, as it is the first to utilize a smartphone application in real patients, relying on photographs for diagnosing wound infections without the direct intervention of healthcare personnel. This innovative approach holds great promise for enhancing the early detection of surgical wound infections and improving patient care.

The main aim of the study was to establish the feasibility and security of telematic monitoring of surgical wounds using the RedScar© application. As a secondary aim, the satisfaction of patients under telematic care with the RedScar© app was evaluated.

## 2. Methods

### 2.1. Study Design

This study utilized a prospective paired cohort study design with a non-randomized, single-blinded approach, where each participant served as their own control. The investigator remained unaware of the application’s recommendations during wound assessments. The study included adult patients undergoing abdominal surgery at a tertiary care hospital in 2021. Patient follow-up care involved a combination of remote telematic monitoring and in-person revisions. The study was conducted following the guidelines of the 2015 Standards for Reporting Diagnostic Accuracy Studies (STARD) Initiative [21], thus ensuring transparency and quality in the presentation of diagnostic accuracy results. The study’s design and protocol underwent thorough review and obtained approval from the Research Ethics Committee of the Balearic Islands (CEI-IB) under reference number IB: 4100/20. Informed consent was obtained from all participants, ensuring patient privacy and data security throughout the study. The study protocol was registered at ClinicalTrials.gov under the identifier NCT05485233.

### 2.2. Sample Size and Recruitment

The sample size was estimated considering that this was a pilot study and the frequency of surgical site infections (SSI). We used a 95% confidence level and an estimated SSI frequency of 5%. To achieve an initial sample size of 30 participants, including a 10% loss rate, we adjusted the desired precision to 0.075 [22].

### 2.3. Inclusion Criteria

We exclusively included adult inpatients who had undergone abdominal surgery with staple closure and who possessed an Android smartphone. Due to the technical limitations of the Android system, only patients whose smartphones had a minimum version of Android 10, with a camera capable of capturing images of a minimum size of 500 × 500 pixels, were accepted.

### 2.4. Exclusion Criteria

Patients lacking access to a compatible smartphone or those unable to use the app correctly were excluded from the study. Additionally, patients unable to undergo personal revisions were excluded.

### 2.5. Identification and Categorization

Potential eligible patients were identified based on our inclusion criteria. We meticulously categorized patients as one of the following: “included”, “missed”, “rejected”, or “excluded”. Detailed records were maintained to track their respective statuses throughout the recruitment process.

### 2.6. Variables

The collected variables included gender, age, ASA risk score, surgery date and type (open/laparoscopic), type of laparotomy (medial incision, subcostal, Pfannenstiel, McBurney, other), discharge date, and length of hospitalization.

### 2.7. Visiting Schedule

Hospitalization and Informed Consent

Patients were discharged post-surgery at the discretion of the responsible physician. Before discharge, participants in the study underwent the same hospitalization protocol as other patients treated at our institution. The study’s protocol was fully explained by the researcher, and written consent was obtained from the patients during this discussion. Subsequently, patients downloaded the RedScar© application (Balearic Island University, Palma, Spain), and the researcher provided a detailed explanation of its functionality, including how to respond to all wound-related questions outlined in Table 1. Patients received a brief training session on how to use the application.

### 2.8. Post-Surgery Follow-Up

**Figure 1:**
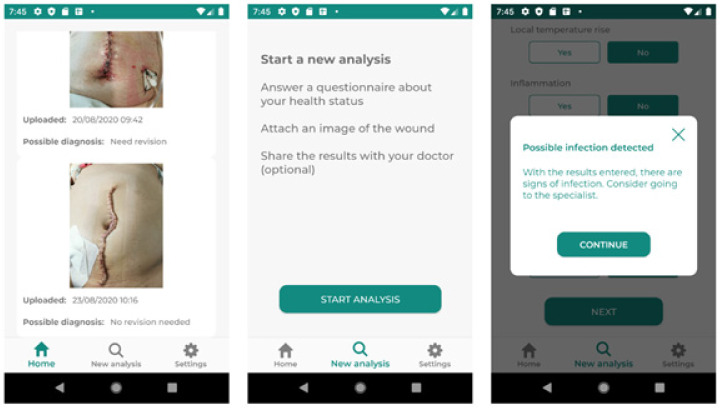
Screenshots of some functionalities of the RedScar© app in the patient interface. From left to right: the history of the images uploaded by the patient, the area to start a new automatic analysis, and finally the result given by the application when a sign of infection has been detected in the analyzed image.

On the 3rd and 10th days following surgery, each patient used the RedScar© application to upload an image of their surgical wound (Figure 1). The application evaluated the risk of wound complications and categorized patients into two groups: those requiring a new consultation due to complications and those with satisfactory wound evolution, eligible for discharge.

#### 2.8.1. Clinical Assessments

On the 3rd and 10th days post-surgery, patients underwent in-person examinations of their surgical wounds by a healthcare professional, either a physician or a nurse. These professionals confirmed the presence or absence of complications and, if deemed necessary, collected microbiological samples to enhance the diagnostic process. The healthcare professionals also completed the questionnaire outlined in Table 1 to compare responses with those provided through the application.

#### 2.8.2. Patient Satisfaction Assessment

Upon concluding the study, a patient satisfaction survey based on the questionnaire developed by Yip et al. [23] and used in similar studies [13] was administered (Table 2). This questionnaire provided valuable insights into patient experiences with the telemedicine-based wound assessment process.

This visiting schedule outlines the comprehensive approach taken to monitor patients’ surgical wounds, combining remote monitoring via the RedScar© application with in-person clinical assessments to ensure the accuracy and reliability of the study’s findings.

### 2.9. Data Analysis Plan

The analysis was conducted on an intention-to-treat basis. Patients removed from the study were not replaced or re-included and were excluded from the analysis. Descriptive analysis of quantitative variables was performed using means and standard deviations or medians and percentiles, with a 95% bilateral confidence interval and range (minimum and maximum) or P50 [P25–P75]. Qualitative variables were summarized in a table that included absolute and relative frequencies. Statistical significance was considered at *p* < 0.05. The IBM^®^ SPSS^®^ Statistics 19 package was used for data analysis.

To measure the agreement between the evaluations provided by two human observers and RedScar©, we employed the kappa statistic as a quantitative measure. To assess concordance, we utilized all the photographs gathered throughout the study, not just those specifically from days 3 and 10. For the intervention, the sensitivity and specificity of the RedScar© application for the detection of wound infection were calculated, and the correlation of clinical diagnoses with the application’s automatic response was compared. The level of redness in infected wound photographs was recorded to improve the infection cut-off point.

Data on patient satisfaction were also collected via a follow-up questionnaire and analyzed separately.

### 2.10. Development of the RedScar© Application

The RedScar© app employs a multifaceted approach to assess surgical wound infections, combining patient-reported data and automatic computer image analysis. The user interface includes a straightforward questionnaire addressing various wound-related aspects, such as pain severity (measured on a visual analog scale, VAS), redness, burning sensation, wound leakage, swelling, liquid secretion, and fever (as outlined in Table 1). To proceed to the next step, which involves uploading a photograph of the wound, patients must respond to all questions in the questionnaire. As shown in González-Hidalgo et al. [24], the application initiates a multistep computerized image analysis process using fuzzy sets, based on gray-scale mathematical morphology (see [20] for more information) and its extension to color images proposed by Bibiloni et al. [25]. Then, by combining this image analysis with the result of the questionnaire, the application autonomously generates a diagnosis without requiring the direct supervision of a healthcare professional (see Image 1). The application has been implemented using Java language and the Android Studio development environment. For the analysis of the image and according to the method proposed in [24], the following repositories implemented by the authors in Python language, which are freely accessible, were used:

- Staple detection [26], which implements the automatic localization of the staples and the wound in the image.
- InPYinting [27], which implements the image reconstruction previously proposed [25].

These repositories have been integrated into the application for image analysis.

All image processing and machine learning methods have been applied and trained, respectively, on the same data set divided into training and testing, randomly prefixed and accessible on redscar.uib.es [28].

### 2.11. Data Protection and Management

Each patient was assigned an identification number to maintain anonymity. A data file was established in which all patient data were anonymized. When a patient was included in the study, a random code was automatically generated by the application, and only the investigator knew the correspondence between the patient and the code.

The application was supported by two servers. The first, a computing server, only received the images, applied algorithms for detecting infection, and returned the value to the device. This service was located at the University of the Balearic Islands (UIB). The second server, which hosted the entire database, was located on Google’s Firebase service. All information was encrypted, and only the researchers involved in the project had access to it. The connection with the RedScar© application was secure, and access was only allowed from the application. Patient information was saved in JSON format (key-value system). Researchers had accounts with the same system, allowing them to be linked with the corresponding patient–physician pairs.

## 3. Results

**Figure 2:**
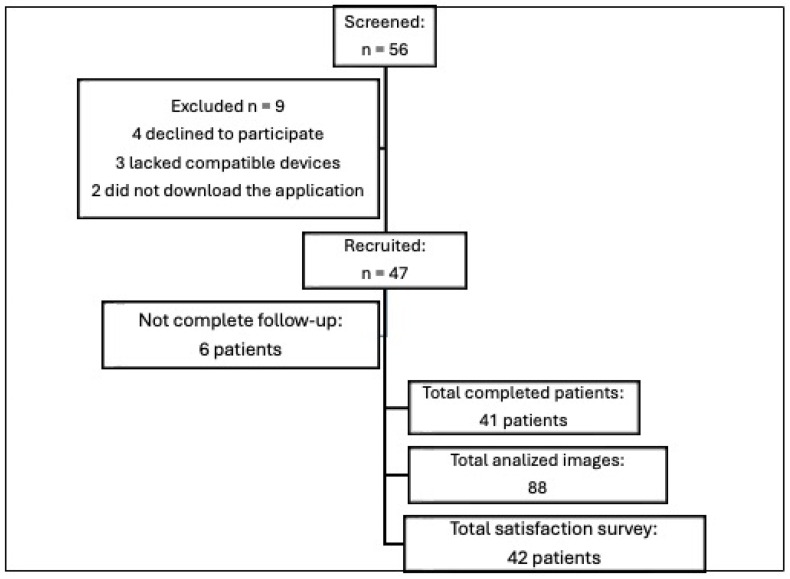
Flow chart of the patients included in the study.

A total of 56 patients met the inclusion criteria. Nine patients were excluded. The exclusion criteria were the absence of a compatible device (three patients), refusal to participate in the study (four patients), and failure to download the mobile application (two patients). Out of the 47 included patients, 41 successfully completed both in-person follow-up and application-based follow-up. The median age within the study cohort was 59 years; 46.8% were females, and 53.2% were males. However, six patients did not complete the study because they either failed to upload the required photo or did not attend the second in-person visit. Nevertheless, the photograph taken on the third postoperative day was utilized in these six cases, resulting in a total of 88 images being analyzed. See Figure 2 for a graphical scheme of the study.

Five (12.19%) patients developed surgical site infections, all of which were detected by the application, resulting in a sensitivity of 100%. The application produced 14 false positives, for which it was indicated that a presential review was needed, but no complications were identified by the clinician. The specificity was 83.13%.

**Figure 3:**
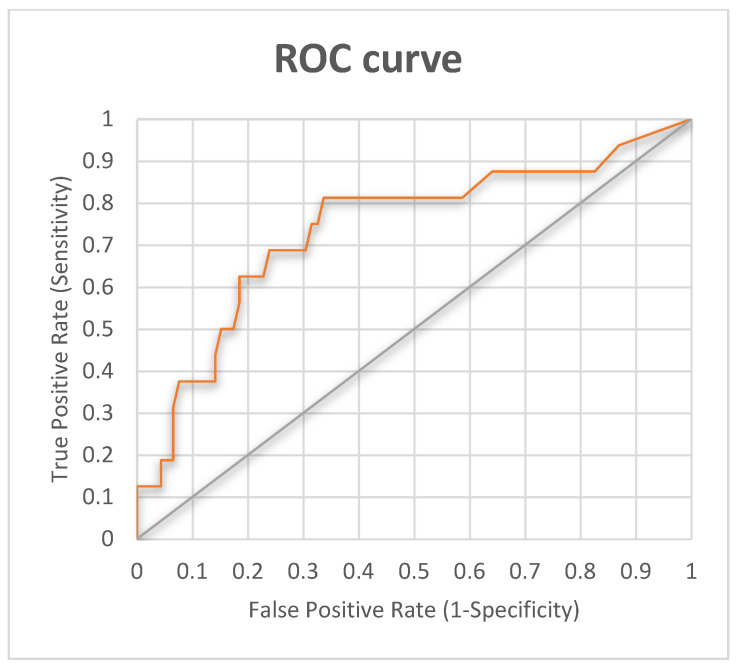
ROC curve for the automatic red proportion detection in the RedScar© app. A cutoff of 0.63 was set.

The detection of infection using automatic image processing was approached in this work from the point of view of the redness of the wound, i.e., the proportion of pixels that had a color close to red. Therefore, determining the optimal threshold for the proportion of red pixels in the image allowed us to detect redness in the wound and to have a criterion for classifying according to the presence of infection. After the analysis of the images, the optimal cutoff point was calculated using the ROC curve, establishing an optimal red proportion value of 0.63. In Figure 3, we display the ROC curve for the automatic red proportion detection.

### 3.1. Concordance

Throughout the study, a total of 363 photographs were captured. These images were used to assess the wound’s progression and evaluate the app’s performance.

The statistical analysis using kappa was conducted on the full set of images (363), which were independently categorized by two researchers. The calculated kappa coefficient of concordance between the application’s results and the observations yielded a value of 0.8171. This value signifies a substantial level of agreement between the human observers and the RedScar© application.

### 3.2. Satisfaction and Patient-Reported Outcomes

A total of 42 patients answered the questionnaire.

**Figure 4:**
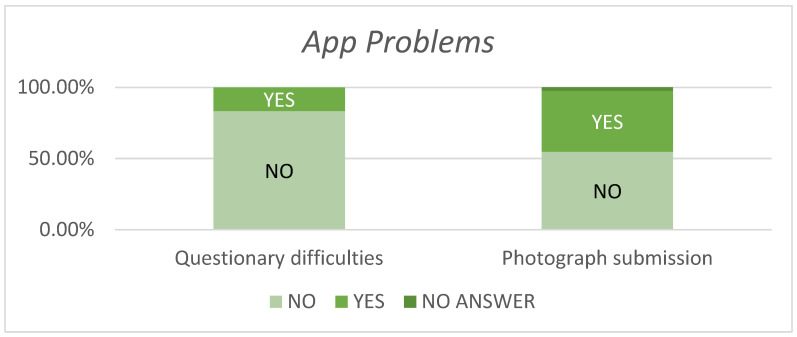
Percentage of patients included in the study who, when using the RedScar© app, encountered some type of problem when completing the questionnaire or attaching an image for automatic analysis.

Concerning photograph submission and analysis, 54.76% of patients experienced no issues, whereas 42.85% encountered app freezes during upload and analysis, and 2.38% did not answer (Figure 4).

The average app time of use was 3 min and 37 s, with a minimum of 1 min of use and a maximum of 15 min, caused by network issues.

**Figure 5:**
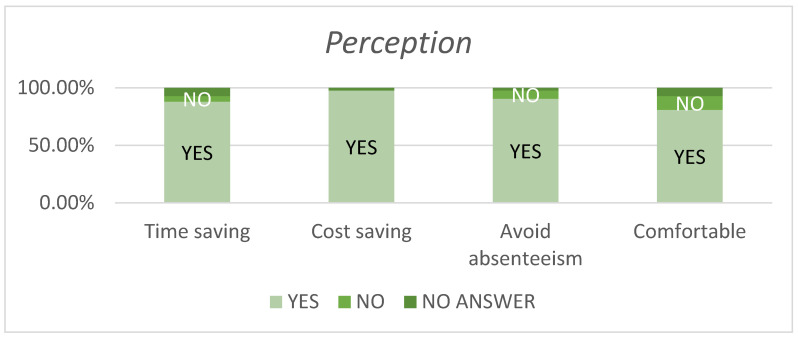
Percentage of patients’ perception of different items on the RedScar application.

Almost all (97.6%) of the respondents believed that telemonitoring would reduce costs, and 90.47% perceived that telemonitoring prevented work/school absenteeism. Additionally, 80.9% found telemonitoring more comfortable (Figure 5).

**Figure 6:**
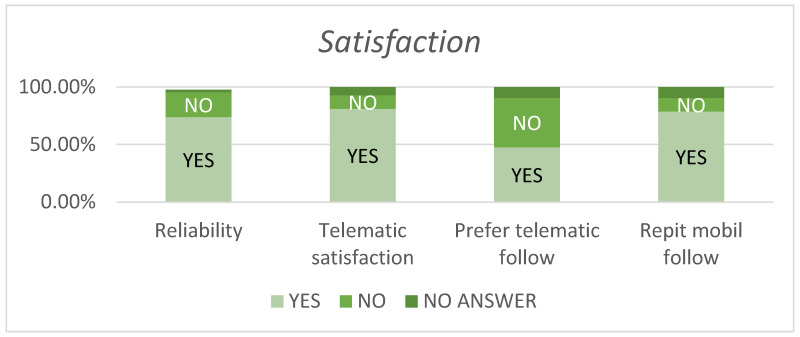
Percentage of patient satisfaction on different items of the RedScar© application.

Regarding reliability, 73.80% of patients found the telemonitoring system trustworthy. Furthermore, 92.85% expressed satisfaction with the provided care, and 80.95% were content with the app’s service quality. Among the 38 patients who responded to the preference question, only 20 patients expressed a preference for telemonitoring. However, the majority (78.57%) stated they would use a similar app for future health monitoring. In terms of app result consultations, 30.95% consulted healthcare professionals based on app indications and 35.71% consulted alternative sources (Figure 6).

## 4. Discussion

The RedScar© application proved to be safe, as no complications derived from telematic follow-up were detected. Telematic follow-up with the RedScar© application was feasible, as most of the patients (85.7%) were able to finish the study. This differs from other studies in which rates of incomplete telemonitoring were high (ranging from 26% to 77%) [19,29,30]. The fact that most of our patients successfully completed the study could be attributed to the user-friendly app and the relatively short 10-day monitoring period [14,17,18].

The kappa value of 0.8171, which measured the agreement between two human blinded observers and RedScar©, indicated a substantial agreement between human observers and the RedScar© application.

To address the absence of professional intervention in both the image acquisition and their subsequent diagnosis, our design aimed for heightened sensitivity, resulting in a 100% final sensitivity outcome. While ensuring the non-underdiagnosis of patients with infection, this comes at the expense of reduced specificity (83.13%), with 16 false-positive detections. Nevertheless, both the sensitivity and specificity are higher than those achieved in other studies [31]. This trade-off means that while a few patients might undergo additional checks unnecessarily, the high sensitivity ensures that most true cases of surgical site infections (SSIs) are detected. In a clinical setting, this is crucial for patient safety and timely intervention. Early detection and treatment of SSIs can significantly improve patient outcomes and reduce complications. This approach is taken to mitigate the risk of false negatives, ensuring that cases of complicated surgical wounds do not go without proper assessment. This helps us avoid the scenario reported by Jia et al. [32], in which out of 81 surgical wound infections, only 66 were diagnosed, and 14 cases escaped detection.

The key innovation of the RedScar© application lies in the automatic analysis of surgical wounds without human intervention. Current telemedicine solutions for wound monitoring typically involve human intervention for diagnosis [19,33]. While various mobile applications exist for diagnosing complicated wounds, most focus on chronic wounds (e.g., diabetic foot ulcers) rather than surgical wounds [34,35]. With RedScar©, patient-uploaded images receive an independent presumptive diagnosis, suggesting the need for clinic visits. This app utilizes only the patient’s smartphone, in contrast to other applications that use costly technologies such as infrared cameras and thermal imaging [17,18,34,36]. The automated analysis eliminates the need for individual clinician assessments, facilitating integration into routine clinical practice, a feature not found in these studies. RedScar© is unique in its fully automated diagnostic process, which does not require direct human supervision. This innovation reduces the burden on healthcare professionals and allows for more scalable and efficient monitoring.

Patient engagement stands as a baseline metric delineated in the proper design and implementation of digital surgical care navigation tools [37]. Our study demonstrates a commendable adherence to this criterion, as evidenced by participants extending their app usage beyond the stipulated duration, utilizing it for more days than required. This behavior may be attributed to its ease of use, immediacy, and user-friendly interface. This circumstance has not been described in other similar papers in the literature, which makes us optimistic about the development of the app. This resulted in a significantly higher number of images than expected, empowering all patients to play an active role in their treatment and recovery. Thanks to the multiple images provided by the patients, a virtual repository of surgical wound photographs was created and made available to other researchers. This repository can be accessed through the website redscar.uib.es (accessed on 10 December 2024) [28].

The great innovation in this app is the diagnostic methodology. There is no app or article that describes the diagnosis of surgical wound images without human intervention. The closest counterparts to our app are those designed for telemedicine-based diagnosis of surgical wound infections, which involve human intervention [33,38,39,40]. In fact, only a study by Scheper et al. [19] comes close to a similar methodology. They employed a mobile phone app with a predetermined score, assessing the likelihood of infection and advising contact with a clinician. However, it is worth noting that the diagnosis relied on the researcher’s evaluation of the patient’s provided photo, rather than the app itself generating the diagnosis.

Similar to the studies analyzed [19,33], our results also reported a prominent level of confidence in telemonitoring (73.80%), although only 47.61% preferred this type of monitoring over in-person follow-up. The observations expressed by some patients in uploading photographs, along with initial malfunctions in the application, could explain this phenomenon. However, 78.57% of the participants would still use a mobile device for monitoring their health status. One area for improvement in future studies is the image upload time, as well as the response time of the application. Additionally, based on the results obtained, enhancing the specificity of the application would help increase patient confidence in RedScar©.

The main limitation of our study is the low wound infection rate, with only five patients (12.19%) experiencing infections. While this aligns with the average infection rate in abdominal surgery [41], further research is currently underway through a multicenter clinical trial with a larger patient population, including those at higher risk of wound infection and in other surgical contexts, such as orthopedic or gynecologic surgeries. This aims to determine the broader applicability of the RedScar© app and better validate these results.

Our findings demonstrate the potential of the RedScar© app to enhance the detection of surgical site infections (SSIs). However, it is crucial to consider potential ethical issues, such as over-reliance on AI in clinical decision-making. Recent studies [42,43] have emphasized that AI can improve prediction accuracy and support surrogate decision-making, but it cannot replicate the nuanced ethical deliberation inherent to human clinicians, and maintaining human oversight is essential to prevent biases and ensure fairness in AI applications in healthcare. Ensuring that AI tools complement human judgment is essential to maintaining high standards of patient care.

An acknowledged constraint in m-health pertains to the age discrepancy in technology adoption [12]. Notably, three out of the four patients who declined participation were aged over 70, while the primary age within the study cohort was 59 years. This observation underscores a propensity for this technology among younger individuals. Addressing this age-related challenge emerges as a promising avenue for bolstering future studies, underscoring the imperative to scrutinize age restrictions and streamline application usage for elderly patients. Studies have shown that older adults often face barriers such as perceived complexity and lack of confidence in using new technologies [44,45]. Ensuring inclusivity in future research will help validate the broader applicability and effectiveness of the RedScar© app across diverse populations.

Currently, the app is only available for Android, resulting in three recruited patients being unable to participate in the study. However, this limitation is in the process of being solved, as the iOS application is under development.

During the study, 42.85% of patients faced challenges when uploading photographs. This may have contributed to not achieving a 100% completion rate. These technical issues have been resolved in the next versions of the app but should be further validated in a subsequent study with a larger patient sample.

Regarding app usage, despite its user-friendliness, some patients struggled to take proper photographs. They failed to center the images correctly, exposed dressings, or used disinfectants with colored substances that hindered the accurate reading of redness. The same difficulties have been reported in other studies [15,46,47], in which the task of taking photographs of the wound was reported as difficult by patients. In future studies, clearer instructions should be provided to patients to ensure optimal results, and the app should be improved regarding these limitations.

## 5. Conclusions

Our study successfully demonstrated the safety and feasibility of telematic follow-up with the RedScar© application. Its high sensitivity suggests its utility in identifying true cases of infection, while the specificity could benefit from further refinement. There was a substantial agreement between the application’s results and human observations, underscoring its promising role in clinical practice. The user-friendly design and the automated diagnosis empowered patients, resulting in a high completion rate and highlighting the application’s key innovation—automatic analysis without human intervention with simple technology. Despite certain limitations, such as a low wound infection rate and technical challenges, our findings emphasize the potential of RedScar© and AI-based apps in routine clinical practice, calling for future studies to validate its efficacy with larger and more diverse patient samples.

## Figures and Tables

**Table 1:** Questionnaire designed to obtain information on the health status of each patient for the RedScar© app.

Question	Response
Visual Analog Scale	0–10 (Scale of pain severity)
Redness around the wound	No (0) Yes (1)
Burning sensation or heat at the wound	No (0) Yes (1)
Wound opening	No (0) Yes (1)
Swelling	No (0) Yes (1)
Liquid secretion	No (0) Yes (1)
Fever	No (0) Yes (1) > 37.7 °C

**Table 2:** Satisfaction questionnaire (17.).

Satisfaction Questionnaire	Answer
1. The information received before participating in the study was adequate.	Yes/No
2. I do not have difficulties with the questionnaire used within the application.	Yes/No
3. I do not have difficulties when taking the photo and uploading it to the mobile application.	Yes/No
4. I have spent less time with telematic follow-up than with personal revision.	Yes/No
5. I think that telematic follow-up is cheaper than the personal revision.	Yes/No
6. I think that telematic review could prevent absenteeism from work/school.	Yes/No
7. Telematic follow-up seems more comfortable to me than follow-up in consultations.	Yes/No
8. I think that telematic monitoring could detect a bad evolution earlier.	Yes/No
9. I think the healthcare provided via telemedicine is consistent.	Yes/No
10. The care received seems adequate to me.	Yes/No
11. Overall, I am satisfied with the quality of service being provided via telemedicine.	Yes/No
12. I would prefer telematic follow-up to face-to-face consultations.	Yes/No
13. I will use telemedicine services again.	Yes/No
14. Following the instructions of the application, have you checked your wound at your health center?	Yes/No, Why?
15. Although the application had ruled out the infection of the wound, have you preferred to check it in person?	Yes/No, Why?
16. Despite using the application, have you consulted any other source (Google-type web search, opinion of close relatives)?	Yes/No, Why?
17. How do you think we can improve the RedScar© application? Leave your comment.	

## Data Availability

Data are available through a virtual repository of surgical wound photographs was created. This repository can be accessed through the website redscar.uib.es (accessed on 10 December 2024) [28].
